# *NPM1* Deletion Is Associated with Gross Chromosomal Rearrangements in Leukemia

**DOI:** 10.1371/journal.pone.0012855

**Published:** 2010-09-21

**Authors:** Roberta La Starza, Caterina Matteucci, Paolo Gorello, Lucia Brandimarte, Valentina Pierini, Barbara Crescenzi, Valeria Nofrini, Roberto Rosati, Enrico Gottardi, Giuseppe Saglio, Antonella Santucci, Laura Berchicci, Francesco Arcioni, Brunangelo Falini, Massimo Fabrizio Martelli, Constantina Sambani, Anna Aventin, Cristina Mecucci

**Affiliations:** 1 Hematology, University of Perugia, Perugia, Italy; 2 Instituto Pelé Pequeno Principe, Faculdades Pequeno Principe, Curitiba, Brazil; 3 Department of Clinical and Biological Sciences, University of Turin, Turin, Italy; 4 Demokritos Cancer Centre, Athens, Greece; 5 Servei de Hematologia, Hospital De La Santa Creu I Sant Pau, Barcelona, Spain; University of Barcelona, Spain

## Abstract

**Background:**

*NPM1* gene at chromosome 5q35 is involved in recurrent translocations in leukemia and lymphoma. It also undergoes mutations in 60% of adult acute myeloid leukemia (AML) cases with normal karyotype. The incidence and significance of *NPM1* deletion in human leukemia have not been elucidated.

**Methodology and Principal Findings:**

Bone marrow samples from 145 patients with myelodysplastic syndromes (MDS) and AML were included in this study. Cytogenetically 43 cases had isolated 5q-, 84 cases had 5q- plus other changes and 18 cases had complex karyotype without 5q deletion. FISH and direct sequencing investigated the *NPM1* gene. *NPM1* deletion was an uncommon event in the “5q- syndrome” but occurred in over 40% of cases with high risk MDS/AML with complex karyotypes and 5q loss. It originated from large 5q chromosome deletions. Simultaneous exon 12 mutations were never found. *NPM1* gene status was related to the pattern of complex cytogenetic aberrations. *NPM1* haploinsufficiency was significantly associated with monosomies (p<0.001) and gross chromosomal rearrangements, i.e., markers, rings, and double minutes (p<0.001), while *NPM1* disomy was associated with structural changes (p = 0.013). Interestingly, in complex karyotypes with 5q- *TP53* deletion and/or mutations are not specifically associated with *NPM1* deletion.

**Conclusions and Significance:**

*NPM1*/5q35 deletion is a consistent event in MDS/AML with a 5q-/-5 in complex karyotypes. *NPM1* deletion and *NPM1* exon 12 mutations appear to be mutually exclusive and are associated with two distinct cytogenetic subsets of MDS and AML.

## Introduction

In humans, nucleophosmin (NPM1) is implicated in the genesis of different cancers [1]. We previously found *NPM1* exon 12 somatic mutations in approximately 60% of adult acute myeloid leukemia (AML) and normal karyotype [2]. NPM1 fusion proteins are also specifically involved: t(5;17)(q35;q21)/*NPM1-RARA* causes acute promyelocytic leukemia; t(2;5)(p23;q35)/*NPM1-ALK* underlies anaplastic large cell lymphoma; t(3;5)(q26;q35)/*NPM1-MLF1* is associated with myelodysplastic syndromes (MDS) and AML with trilineage dysplasia and poor prognosis (http://AtlasGeneticsOncology.org). In the last cytogenetic subgroup of MDS/AML a cryptic loss of *NPM1* gene may occur at the 5q35 breakpoint of the translocation [3]. Despite of this sporadic observation the role of *NPM1* deletion in hematological malignancies remains to be elucidated. In human leukemia an interstitial isolated deletion at chromosome 5q identifies a myelodysplastic syndrome with a benign clinical course (the “5q- syndrome”) [4]. Moreover a partial or complete 5q loss (5q-/-5) may occur as part of a complex karyotype in high risk MDS/AML, including cases arising after radio- and/or chemo-therapy [5]. Chromosomes 5q- are heterogeneous in size as deleted segments vary in extension and have different centromeric and telomeric breakpoints [6], [7]. Experimental evidences indicate that the 5q- syndrome originates from haploinsufficiency of more than one critical pathogenetic gene included in the hemizygous chromosome deletion [8]–[12]. Thus identification of genes that go lost in different 5q- chromosomes of MDS and AML is very important to understand the pathophysiology of the associated malignant disorders. Focusing on *NPM1* gene we investigated a large series of MDS and AML with and without a 5q deletion.

## Materials and Methods

### Patient inclusion criteria

AML or MDS and one of the following abnormal karyotypes, according to the ISCN [13]: isolated 5q-; 5q-/-5 plus one or more additional changes (non-isolated); complex karyotypes without 5q-/-5. 145 patients (60 males; 85 females; age range 9–94 years; 71 with MDS and 74 with AML) were recruited from the Hematological Institutions, University of Perugia, Italy, Sant Pau Hospital, Barcelona, Spain, and the Demokritos Cancer Centre, Athens, Greece.

### FISH

Protocols have already been described [14]. Commercial FISH probes were provided by Abbott/Vysis (Downers Grove, IL, USA): LSI EGR1/D5S721, D5S23 dual color probe; LSI CSF1R (5q33–q34) spectrum orange/D5S721, D5S23 spectrum green; LSI c-MYC dual color break apart rearrangement probe; LSI 13 (RB1) 13q14 spectrum orange probe. Caltech DNA clones CTC-286C20 (*FGFR4*/5q35.2), CTC-549A4 (*NSD1*/5q35.3), CTD-2131H15 (*3′CTNNA1*/5q31), CTD-2324F6 (*5′CTNNA1*/5q31), CTD-2342K5 (*BRCA2*/13q12), and CTD-3199J23 (*BRCA1*/17q21) were purchased from Invitrogen (Carlsbad, CA, USA). Other DNA clones were selected from the RPCI libraries (http://www.ncbi.nlm.gov/project/mapview, Build 37.1), provided by Mariano Rocchi, Department of Genetics and Microbiology, University of Bari, Italy and grown, labelled, and validated at the Cytogenetics and Molecular Genetics Laboratory, Hematology Department, University of Perugia, Italy. Clones mapping at 5q11.2–5q14.1 listed from centromere to telomere: RP11-266N13, RP11-489L13, RP11-298P6, RP11-79C20, RP11-170N5, RP11-195G20, RP11-633M1, RP11-195E2, RP11-771B3, RP11-79P5, RP5-910M8, RP11-229C3, RP11-469J18, RP11-996M9, RP11-168A11, RP11-80K5, RP11-1089B2, and RP11-885P10. Gene-specific RPCI clones: RP11-89G4 (*IRF1*/5q31), RP11-946D14 (*RPS14*/5q33), RP11-204L7 (*3′SPARC*/5q33), RP11-642K17 (*5′SPARC*/5q33), RP11-117L6 (*NPM1*/5q35.1), RP1-240G13 (5qter subtelomeric probe), RP11-480O8 (*BUB1A*/2q14), RP11-383G6 (*3′ATR*/3q23), RP11-427D1 (*5′ATR*/3q23), RP11-669K4 (*FBW7*/4q31), RP11-149I2 (*CDKN2A*/B/9p21), RP11-380G5 (*PTEN*/10q23), RP11-241D13 (*ATM*/11q22), RP11-880O16 (*CHEK1*/11q24), RP11-1137N1 (*MDM2*/12q15), RP11-248I17 (*BUB1B*/15q15), RP11-436C9 (*3′CHEK2*/22q12), RP11-444G7 (*5′CHEK2*/22q12).

### *NPM1* and *TP53* Mutational Analysis

Exon 12 *NPM1* mutations were studied by direct sequencing of PCR products from genomic DNA using the following primers: NPM1_ex12for 5′-ATGTCTATGAAGTGTTGTGGTTCC-3′ and NPM1_ex12rev 5′- CAGGCATTTTGGACAACACA-3′. *TP53* gene (NC_000017.10, 7590863.7571720) mutations on exons 2-12 (**Table 1**) were studied using PCR-based denaturing HPLC using a WAVE-MD™ System (Transgenomic, Omaha, NE) equipped with a DNASep Cartridge. PCR assays were performed in a volume of 25 µl, containing 100 ng of genomic DNA, 6 pmol of forward and reverse primer, 200 mM dNTPs, 0.3 U of AmpliTaq Gold (Life Technologies Corp., Carlsbad, CA, USA). Gradient elution and melting temperature conditions were established by Wave-Maker Navigator version 1.7 software (Transgenomic). Bidirectional sequencing was performed on samples with abnormal chromatographs using ABI prism 3130 (Life Technologies). Missense and frameshift mutations were detected using Finch TV version 1.4.0 and described according to CCDS 11118.1 (NM_0000546.4).

**Table 1:** Primers used to amplify *TP53* coding exons (NC_000017.10).

*TP53* Exon	Primer	Sequence
2	P53_ex2FP53_ex2TGFP53_ex2R	5′-TTTTCCTCTTGCAGCAGCCA-3′ 5′-CCAGGTGACCCAGGGTTGGAAG-3′ 5′-CAAGAGCAGAAAGTCAGTCC-3′
3	P53_ex3FP53_ex3R	5′-AGACCTGTGGGAAGCGAAAA-3′ 5′-GGGACAGCATCAAATCATCC-3′
2–3	P53_ex2-3FP53_ex2-3R	5′-TGCCTTCCGGGTCACTGCC-3′ 5′-AGCCCTCCAGGTCCCCAGCC-3′
4	P53_ex4FP53_ex4AFP53_ex4RP53_ex4AR	5′-ATCTACAGTCCCCCTTGCCG-3′ 5′-ACCTGGTCCTCTGACTGCTC-3′ 5′-GCAACTGACCGTGCAAGTCA-3′ 5′-GCCAAAGGGTGAAGAGGAAT-3′
5	P53_ex5FP53_ex5AFP53_ex5RP53_ex5AR	5′-GCTGCCGTGTTCCAGTTG-3′ 5′-GCTTTATCTGTTCACTTGTGCC-3′ 5′-ACCAGCCCTGTCGTCTCTC-3′ 5′-AGCCCTGTCGTCTCTCCAG-3′
6	P53_ex6FP53_ex6R	5′-CCAGGCCTCTGATTCCTCAC-3′ 5′-GCCCCCCTACTGCTCACC-3′
7	P53_ex7FP53_ex7RP53_ex7AR	5′-GCCACAGGTCTCCCCAAG-3′ 5′-TGTGCAGGGTGGCAAGTG-3′ 5′-TGCAGGGTGGCAAGTGGCTC-3′
8	P53_ex8FP53_ex8R	5′-TGCCTCTTGCTTCTCTTTTCC-3′ 5′-GGCATAACTGCACCCTTGG-3′
9	P53_ex9FP53_ex9R	5′-GCGGTGGAGGAGACCAAG-3′ 5′-GCTACAACCAGGAGCCATTG-3′
10	P53_ex10FP53_ex10R	5′-GGCAGTGATGCCTCAAAGAC-3′ 5′-CTAGGCTAAGCTATGATGTTCC-3′
11	P53_ex11FP53_ex11AFP53_ex11DFP53_ex11RP53_ex11ARP53_ex11BR	5′-CCCCCTCCTCTGTTGCTGC-3′ 5′-TACTTCTCCCCCTCCTCTG-3′ 5′-GAACCATCTTTTAACTCAGGTAC-3′ 5′-GGCAGGGGAGTAGGGCCAG-3′ 5′-GAAGGCAGGATGAGAATGGA-3′ 5′-AGCTGCCTTTGACCATGAAG-3′
12	P53_ex12FP53_ex12AFP53_ex12BFP53_ex12RP53_ex12AR	5′-CACTCATGTGATGTCATCTCTC-3′ 5′-CTCACTCATGTGATGTCATCT-3′ 5′-CTCTGAGGTGCTCAGTAAAC-3′ 5′-GCTGTCAGTGGGGAACAAGA-3′ 5′-GCAAGCAAGGGTTCAAAGAC-3′

### *NPM1* expression by Real Time Quantitative PCR

RT-qPCR was performed on cryopreserved bone marrow RNA samples from 9 patients with *NPM1*+/- and 5q-, 10 patients with *NPM*1+/+ and 5q-, 5 patients with *NPM1*+/+ without 5q- and 11 healthy controls. Total RNA was extracted using Trizol (Invitrogen) according to the manufacturer's protocol with minor modifications. Two micrograms of total RNA were treated with 4 Units of Dnase I (Deoxyribonuclease I Amplification Grade, Sigma) in a total volume of 11 µl. One microgram of treated RNA was reverse transcribed to single strand cDNA according to the EAC protocol [15] and 1 µg was used in a control reaction (-RT) using the same procedure without the M-MLV reverse transcriptase enzyme (Perkin Elmer Applied Biosystems, Foster City, USA). The RT-qPCR mixture reaction contained 12.5 µl Taq Man Universal PCR Master Mix (Life Technologies), 300 nM primers, 200 nM probe and 5 µl cDNA (1/10 of RT product), in a total volume of 25 µl. Amplification conditions were: 2 minutes at 50°C, 10 minutes at 95°C followed by 40 cycles at 95°C for 15 seconds and at 60°C for 1 minute [16]. RT-qPCR reactions and fluorescence measurements were performed on an ABI PRISM 7700 Sequence Detection System (Life Technologies). Gene expression levels of *NPM1* and *ABL1* were detected using specific assays on Demand (Life Technologies) following the manufacturer's instructions: ID: Hs02339479_g1 (exon 4–5) for *NPM1* and ID: Hs00245445_m1 (exon 3–4) for *ABL1*. RT-qPCR reactions were performed in quadruplicate for *NPM1* and in duplicate for *ABL1. NPM1* transcript levels were quantified relative to endogenous *ABL1* and expressed as 2^−ΔΔCt^ [17]. Universal Human Reference RNA (Stratagene, La Jolla, CA, USA) was used as calibrator in all experiments. A threshold value of 0.1 was used throughout the study.

### Statistical analysis

Inter-group differences were analyzed by non parametric tests using first analysis of variance (ANOVA) with the Kruskal-Wallis test to compare three groups and the Mann-Whitney U test to compare two. If significant, multiple comparisons were carried out using the Mann-Whitney U test with the Bonferroni correction (i.e. 0.05/numbers of comparisons). The multivariable Poisson regression model analyzed the adjusted effect of *NPM1* and *TP53* on gross chromosomal changes and monosomies.

Contingency tables studied associations among categorical variables which were analyzed by Fisher's Exact test for 2X2 tables or the chi-square test. Unless otherwise indicated, significance was set at <0.05. SPSS version 17.0 software (Chicago, IL) was used for statistical analysis.

### Ethics statement

This study was carried out solely on encoded archival samples that were originally taken for diagnostic purposes. The residual of these samples was used after all diagnostic procedures had been completed. Our specific study was approved by the Institutional Review Board of the Hematology Department of the University of Perugia (IRB 00003450) which guidelines allow the use of anonymous residual diagnostic samples for research purposes. Written informed consent was obtained. The “Demokritos” Cancer Research Centre Ethics Committee (Athens, Greece) allows the use of anonymous or encoded residual diagnostic samples for research purposes, unless the patient expresses its refusal to participate in research studies. The authors declare that all samples used in this study were encoded anonymously and that none of the patients had explicitly refused to participate in research.

## Results and Discussion

One hundred forty five patients with MDS/AML (60 males and 85 females; median age = 68 years, range 9–94) were included in the study (**Table S1**).

The mono-allelic *NPM1* deletion was a very infrequent lesion in the “5q- syndrome” because it was not detected in 42/43 cases. It identified two molecular sub-groups, here named *NPM1*+/− and *NPM1*+/+, since it was present in 38/84 (45%) cases with complex karyotypes and 5q-. Interestingly Lessard et al. [18] demonstrated loss of the 5q35 chromosome band in 30% of de novo and therapy related AML. *NPM1* loss may have occurred as an early step in MDS/AML as it remained stably mono-allelic in 4 *NPM1+/−* patients and did not develop in 6 *NPM1+/+* over a median of 9 months follow-up (range: 2–156 months) (**Table 2**).

**Table 2:** **FISH:** *NPM1* status over time (median 9 months; range 2–156) in 10 adult patients with MDS/AML.

No.	Diagnosis	Karyotype	Follow-up (months)/Disease status	Karyotype/*NPM1*
		**Non-isolated 5q- ** ***NPM1*** ** +/+**		
**45**	RA	47,XX,del(5q),+21	+156/AML	46,idem,-7,t(12;22(p13;q11))/U
**48**	RAEB	46,XX,add(11)(q),del(5q)/46,XX	+24/RAEB	U/U
**57**	AML	46,XX,del(5q),t(2;3)(p21;q26)	+4/AML	45,idem,−7/U
**72**	AML	45–46,XX,t(3;11)(p21;q23),del(5)(q13q31),−7,+8,del(12)(p13),add(16)(p13),add(17)(q),−18,+mar/46,XX	+13/2^nd^ relapse	U/U
**73**	RAEB	46,XX,del(5)(q13q33),del(7)(q22q32),del(7)(q22q32),der(20)/46,idem,add(7)(q36)	+13/MDS-U	U/U
**76**	AML	41–49,XY,del(2p),der(3)(p21),−5,del(8)(q22),der(13;15)(q10;q10),−17,−18,+1−6 mar/46,XY	+5/2^nd^ relapse	U/U
		**Non-isolated 5q- ** ***NPM1*** ** +/−**		
**93**	AML	42–44,XX,−5,add(6)(p21),+8,1–3mar	+9/1^st^ relapse	n.d./U
**110**	AML	44,XY,−2,−3,−5,−7,−13,−17,+mar1,+mar2,+mar3/46,XY	+9/1^st^ relapse	n.d./U
**121**	RAEB	40–48,XY,t(1;3)(p32;p21),−5,−7,−13,−18,−20,−22,+1−6 mar/46,XY	+2/RAEB	n.d./U
**122**	AML	40−43,XY,add(1)(q),−4,del(5)(q13q33),−7,−9,−12,−17,−20,+2-6mar	+9/resistant	n.d./U

Patient no. (see Supplementary Table 1); AML, acute myeloid leukemia; RAEB, refractory anemia with excess of blasts; RA, refractory anemia; *NPM1*+/+ no monoallelic *NPM1* deletion; *NPM1*+/− monoallelic *NPM1* deletion; U, unchanged; n.d., not done.

**Figure 1:**
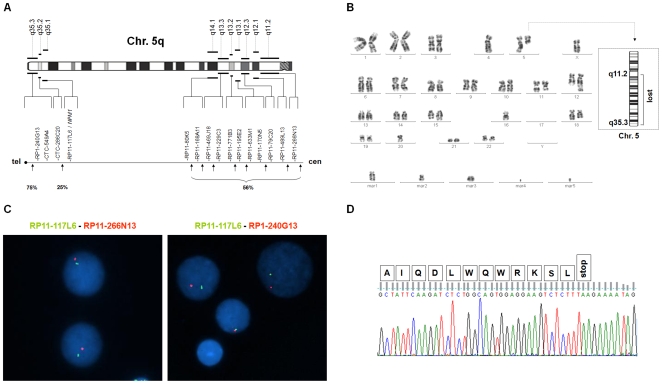
FISH, cytogenetics and mutational analysis. **A**) Results of FISH in 38 patients with MDS/AML and *NPM1* monoallelic deletion (*NPM1*+/−): schema of 5q breakpoints. Upper: ideogram of the long arm of chromosome 5 in G banding. Lower: genomic clones at proximal (q11.2– q14.1 sub-bands) and distal (q35.1–q35.3 sub-bands) breakpoints with percentages of cases. **B**) G-banded karyotype of a representative case (n.103, Supporting Table S1) showed a complex karyotype including monosomies, unbalanced translocations, five markers, and a very small deleted chromosome 5 corresponding to the largest 5q deletion including *NPM1* with breakpoints at q11.2 and q35.3 (Right ideogram). **C**) FISH of case n.103: concomitant deletion of RP11-117L6/*NPM1* (green) and RP11-266N13 (red) indicate centromeric breakpoint (left) and RP1-240G13/subtelomeric sequences (red) indicate telomeric breakpoint (right). Only one copy of each clone is present. **D**) Gene sequencing of case n.103 shows no *NPM1* exon 12 mutation in the non-deleted chromosome 5. The last 12 amino acids encoded by exon 12 of wild type *NPM1* (NM_002520) are annotated on top of the sequence.

The *NPM1* loss was not cryptic since it was absent in 18 patients with complex karyotypes without 5q- (**Table S1**). Chromosome 5q breakpoints were distributed over a wide chromosomal area within genomic regions which extended beyond the isolated 5q deletion breakpoints. In *NPM1*+/− patients telomeric breakpoints clustered between *NPM1* and *FGFR4* (approximately 5.7 megabases) in 4/16 cases and included subtelomeric sequences in 12 (75%) (**Figure 1A,B,C** **,** and **Table S1**). Centromeric breakpoints involved chromosome bands from q11.2 to q14.1 in more than 50% and were proximal to RP11-80K5 (**Figure 1A,B,C** **,** and **Table S1**). Notably in cases with isolated 5q deletions centromeric breakpoints always fell distally to RP11-80K5 (**Figure 1A** and **Table S1**).

**Figure 2:**
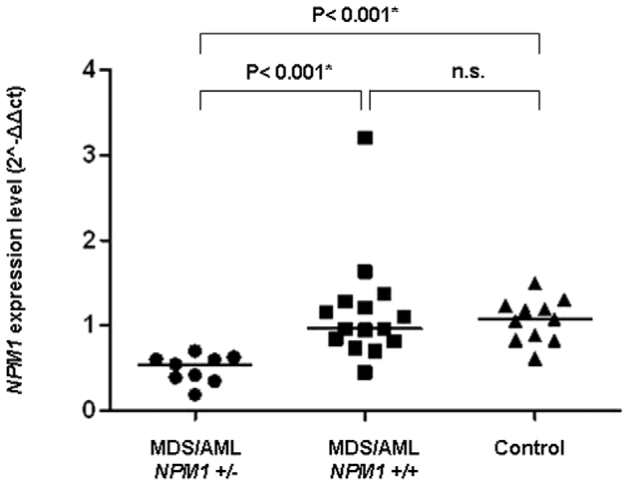
RT-qPCR and *NPM1* gene expression. *NPM1* expression in 9 patients with MDS/AML *NPM1*+/− (median: 0.5443; range: 0.18÷0.70), in 15 with MDS/AML *NPM1*+/+ (median: 0.96; range: 0.44÷3.20) and in 11 healthy controls (median 1.07; range 0.61÷1.50). Horizontal lines indicate median *NPM1* expression in each group. Y axis  = 2∧−ΔΔCt value (*NPM1* gene expression level normalized to endogenous *ABL1* gene) ΔCt = Ct *_NPM1_* −Ct _ABL1_, ΔΔCt =  ΔCt _sample_−ΔCt_calibrator_. (P values according to the Mann-Whitney U test and Bonferroni correction); n.s. =  non significant.

Exon 12 mutations were not found in this study indicating that monoallelic *NPM1* deletion and heterozygous mutation can not occur at the same time (**Figure 1D** and **Table S1**). Furthermore, the *NPM1* deletion corresponded to reduced *NPM1* expression in MDS/AML cells which was significantly lower in *NPM1*+/− cases than in the *NPM1*+/+ (P<0.001) and normal controls (P<0.001) (**Figure 2**). Notably, NPM1 is a multifunctional protein which regulates ribosome biogenesis, TP53 protein activity and stability, ARF protein stability and localization, and DNA integrity [1], [19], [20]. In keeping with evidence from murine models [21], in which *NPM1* loss induced morphological signs of MDS, favored *c-MYC* lymphomagenesis and underlay chromosomal instability, chromosome rearrangements constituting complex karyotypes were significantly different in *NPM1*+/− and *NPM1*+/+ patients. In *NPM1*+/+ structural chromosome rearrangements (translocations; deletions; insertions; inversions) were significantly more frequent (median 3, range 0–7 vs median 1.5, range 0–5; P = 0.013) (**Table S2**) but in the *NPM1*+/− group gross chromosomal changes (markers i.e. derivative chromosomes unclassified by karyotyping, rings, and double minutes) were significantly more common (median 3, range 0–9 vs median 0, range 0–6; P<0.001) (**Table S2**). Aneuploidy was present in 47/64 *NPM1*+/+ patients (73%) and in 38/38 *NPM1*+/− patients (100%) (P<0.001). Significantly more monosomies were found in the *NPM1*+/− subgroup (median 2, range 0–8 vs median 1, range 0–6; P<0.001) (**Table S2**), with the most frequent, monosomy 7, being detected in 18/38 (47%) of *NPM1*+/− and in 11/64 (17%) of *NPM1*+/+ (P = 0.002).

Since *NPM1* is related to *TP53* [19], , a master gene of chromosome stability that is frequently involved in MDS/AML with complex karyotype [24], 57 cases from this series were investigated (**Table 3** and **TableS1**). Univariate analysis on *TP53* and chromosome rearrangements showed a significant association between the number of monosomies and *TP53* deletion and/or mutation (median 2 range 0−8 vs median 0 range 0−3; P = 0.006) (**Table S3**). However, both *NPM1* and *TP53* gene status maintained significant association with monosomies in a multivariate analysis (P = 0.014 and P = 0.013, respectively) (Poisson regression model), suggesting each gene plays an independent role, but may interact synergistically, in determining chromosomal abnormalities.

**Table 3:** *TP53* monoallelic deletion and/or mutations in 49/57 patients with MDS/AML and non-isolated 5q-.

	*TP53*_del_	*TP53*_mut_	*TP53*_del_/*TP53*_mut_
***NPM1*** **+/+**	11	7	5
***NPM1*** **+/−**	11	11	4

del, monoallelic deletion; mut, mutation.

**Figure 3:**
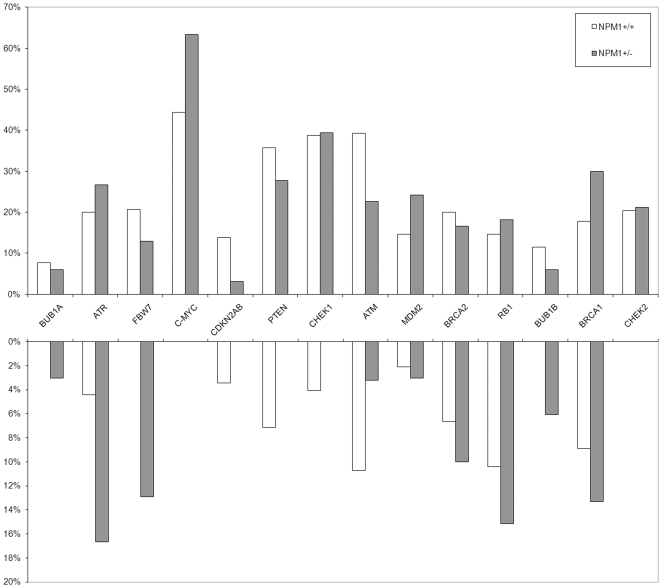
FISH investigations on clones encompassing 14 genes that are putatively involved in genomic stability. Gains (upper) and losses (lower) in *NPM1*+/+ patients (white columns) and *NPM1*+/− (grey columns).

*NPM1* loss was not associated with copy number variations of 14 genes other than *TP53* that are putatively involved in the complex regulatory network of genetic stability. Gains and losses of clones containing *BUB1A, ATR, FBW7, c-MYC, CDKN2A-2B, PTEN, CHEK1, ATM, MDM2, BRCA2, RB1, BUB1B, BRCA, CHEK2* were equally distributed in *NPM1*+/+ and *NPM1*+/− cases (**Figure 3**), suggesting that whatever roles these genes play in MDS/AML with complex karyotypes, they are independent of the *NPM1* deletion.

**Figure 4:**
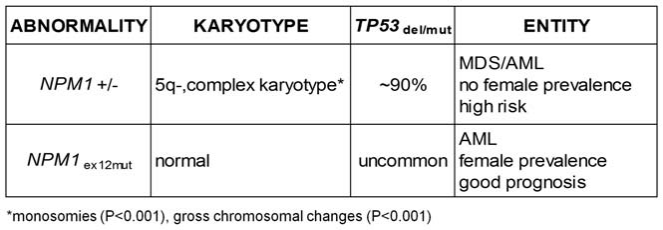
Mutually exclusive NPM1 abnormalities indicate different leukemogenic pathways.

In conclusion these findings prove that haploinsufficiency of the *NPM1* gene, is present in more than 40% of patients with high risk MDS/AML characterised by chromosome 5q- and complex karyotypes. *NPM1* haploinsufficiency and mutation appear to exert very different effects (**Figure 4**) as patients with *NPM1* mutated-AML have normal karyotype, rare *TP53* involvement, good prognosis if *FLT3-*ITD mutations are absent, and a predominance of females [2], [25], [26]. *NPM1* haploinsufficiency was not linked to female gender (**Table S4**). It significantly reduced *NPM1* expression and, in synergy but independently of *TP53* deletion and/or mutation, was associated with a strong tendency to perturb chromosomal stability. Consequently *NPM1* gene status appears to indicate diverse genetic backgrounds in two different myeloid leukemias.

## Supporting Information

Table S1 Demographics, clinical findings, results of cytogenetics, FISH and mutational analysis in 145 patients with MDS/AML. Footnotes: M, male; F, female; RAEB, refractory anemia with excess of blasts; RA, refractory anemia; AML, acute myeloid leukemia; RCMD, refractory cytopenia with multilineage dysplasia; MDS-U, myelodysplastic syndrome, unclassifiable; RARS, refractory anemia with ringed sideroblasts; ex, exon; DEL, monoallelic deletion; NL, normal hybridization pattern; GAIN, ≥3 hybridization signals; (a) cases with *NPM1* RT-qPCR analysis; (b) copy number >5.
(0.12 MB XLS)

Table S2 Distribution of markers, monosomies, structural aberrations, and trisomies in *NPM1*+/+ (0) and *NPM1*+/− (1) cases (Mann-Whitney U Test).
(0.47 MB DOC)

Table S3 Distribution of markers, monosomies, structural aberrations, and trisomies according to the *TP53* status (0 =  no deletion or mutation; 1 =  deletion and/or mutation) (Mann-Whitney U Test).
(0.47 MB DOC)

Table S4 Sex distribution between in *NPM1*+/+ and *NPM1*+/− groups. Sex (males = 0, females = 1) distribution between in *NPM1*+/+ (0) and *NPM1*+/− (1) groups.
(0.47 MB DOC)
