# Supplementary material for: NPM1 Deletion Is Associated with Gross Chromosomal Rearrangements in Leukemia
Source: PLoS One. 2010 Sep 21;5(9):e12855. doi: 10.1371/journal.pone.0012855 (PMC2943467; doi:10.1371/journal.pone.0012855)
Supplement: Table S2 — Distribution of markers, monosomies, structural aberrations, and trisomies in NPM1+/+ (0) and NPM1+/− (1) cases (Mann-Whitney U Test). (0.47 MB DOC) [file pone.0012855.s002.doc]

**Table S2**. Distribution of markers, monosomies, structural aberrations, and trisomies in *NPM1*+/+ (0) and *NPM1*+/- (1) cases (Mann-Whitney U Test).

| **Ranks** | | | | |
| --- | --- | --- | --- | --- |
|  | *NPM*1 | number of cases | median range | Rank sum |
| markers | 0 | 64 | 41.50 | 2656.00 |
|  | 1 | 38 | 68.34 | 2597.00 |
|  | Total | 102 |  |  |
| monosomies | 0 | 64 | 42.84 | 2742.00 |
|  | 1 | 38 | 66.08 | 2511.00 |
|  | Total | 102 |  |  |
| structural aberrations | 0 | 64 | 56.94 | 3644.00 |
|  | 1 | 38 | 42.34 | 1609.00 |
|  | Total | 102 |  |  |
| trisomies | 0 | 64 | 51.13 | 3272.00 |
|  | 1 | 38 | 52.13 | 1981.00 |
|  | Total | 102 |  |  |

| **Test^a^** | | | | |
| --- | --- | --- | --- | --- |
|  | markers | monosomies | structural aberrations | trisomies |
| Mann-Whitney U | 576.000 | 662.000 | 868.000 | 1192.000 |
| Wilcoxon W | 2656.000 | 2742.000 | 1609,000 | 3272.000 |
| Z | -4.592 | -3.938 | -2.482 | -.194 |
| 2-sided Asymp Sig. | .000 | .000 | .013 | .846 |
| a. Grouping variable: *NPM1* | | | | |
