# Supplementary material for: NPM1 Deletion Is Associated with Gross Chromosomal Rearrangements in Leukemia
Source: PLoS One. 2010 Sep 21;5(9):e12855. doi: 10.1371/journal.pone.0012855 (PMC2943467; doi:10.1371/journal.pone.0012855)
Supplement: Table S3 — Distribution of markers, monosomies, structural aberrations, and trisomies according to the TP53 status (0 = no deletion or mutation; 1 = deletion and/or mutation) (Mann-Whitney U Test). (0.47 MB DOC) [file pone.0012855.s003.doc]

**Table S3.** Distribution of markers, monosomies, structural aberrations, and trisomies according to the *TP53* status (0= no deletion or mutation; 1= deletion and/or mutation) (Mann-Whitney U Test).

| **Ranks** | | | | |
| --- | --- | --- | --- | --- |
|  | abnormal *TP53* | number of cases | mean rank | rank sum |
| markers | 0 | 8 | 20.44 | 163.50 |
|  | 1 | 49 | 30.40 | 1489.50 |
|  | Total | 57 |  |  |
| monosomies | 0 | 8 | 14.50 | 116.00 |
|  | 1 | 49 | 31.37 | 1537.00 |
|  | Total | 57 |  |  |
| structural aberrations | 0 | 8 | 25.50 | 204.00 |
|  | 1 | 49 | 29.57 | 1449.00 |
|  | Total | 57 |  |  |
| trisomies | 0 | 8 | 27.38 | 219.00 |
|  | 1 | 49 | 29.27 | 1434.00 |
|  | Total | 57 |  |  |

| **Test** | | | | |
| --- | --- | --- | --- | --- |
|  | markers | monosomies | structural aberrations | trisomies |
| Mann-Whitney U | 127.500 | 80.000 | 168.000 | 183.000 |
| Wilcoxon W | 163.500 | 116.000 | 204.000 | 219.000 |
| Z | -1.612 | -2.709 | -.669 | -.364 |
| 2-sided Asymp Sig. | .107 | .007 | .504 | .716 |
| Significance [2*(1-tailed significance)] | .117^a^ | .006^a^ | .535^a^ | .778^a^ |
| a. Not corrected for equal values. | | | | |
| b. Grouping variable: abnormal *TP53.* | | | | |
