# Supplementary material for: NPM1 Deletion Is Associated with Gross Chromosomal Rearrangements in Leukemia
Source: PLoS One. 2010 Sep 21;5(9):e12855. doi: 10.1371/journal.pone.0012855 (PMC2943467; doi:10.1371/journal.pone.0012855)
Supplement: Table S4 — Sex distribution between in NPM1+/+ and NPM1+/− groups. Sex (males = 0, females = 1) distribution between in NPM1+/+ (0) and NPM1+/− (1) groups. (0.47 MB DOC) [file pone.0012855.s004.doc]

**Table S4.** Sex (males=0, females=1) distribution between in *NPM1*+/+ (0) and *NPM1*+/- (1) groups.

| **Contingency table** | | | | | |
| --- | --- | --- | --- | --- | --- |
|  | | | *NPM1* | | Total |
|  |  |  | 0 | 1 |  |
| SEX | 0 | Count | 24 | 23 | 47 |
|  |  | % SEX | 51.1% | 48.9% | 100.0% |
|  |  | % *NPM1* | 37.5% | 60.5% | 46.1% |
|  | 1 | Count | 40 | 15 | 55 |
|  |  | % SEX | 72.7% | 27.3% | 100.0% |
|  |  | % *NPM1* | 62.5% | 39.5% | 53.9% |
| Total | | Count | 64 | 38 | 102 |
|  |  | % *SEX* | 62.7% | 37.3% | 100.0% |
|  |  | % *NPM1* | 100.0% | 100.0% | 100.0% |

| **Chi-square** | | | | | |
| --- | --- | --- | --- | --- | --- |
|  | Value | df | Asymp.Sig  (2-sided) | Exact Sig. (2-sided) | Exact Sig. (1-sided) |
| Pearson Chi-square | 5.088^a^ | 1 | .024 |  |  |
| Continuity^b^ | 4.204 | 1 | .040 |  |  |
| Likelihood ratio | 5.112 | 1 | .024 |  |  |
| Fisher’s exact test |  |  |  | .039 | .020 |
| Linear-linear association | 5.038 | 1 | .025 |  |  |
| No of valid cases | 102 |  |  |  |  |
| a. 0 cells (.0%) have an expected count less than 5. The minimum expected count is 7.51. | | | | | |
| b. Computed only for a 2x2 table. | | | | | |
